# Extracting the morphology of gold bipyramids from small-angle X-ray scattering experiments via form factor modelling

**DOI:** 10.1107/S1600576722011669

**Published:** 2023-02-01

**Authors:** Jieli Lyu, Claire Goldmann, Cyrille Hamon, Doru Constantin

**Affiliations:** a Laboratoire de Physique des Solides, CNRS, Université Paris-Sud, Université Paris-Saclay, 91405 Orsay Cedex, France; b Institut Charles Sadron, CNRS and Université de Strasbourg, 67034 Strasbourg, France; Argonne National Laboratory, USA

**Keywords:** small-angle X-ray scattering, SAXS, colloids, bipyramids, form factors, transmission electron microscopy, TEM

## Abstract

Accurate shape description is a challenge in materials science. Small-angle X-ray scattering (SAXS) can provide the shape, size and polydispersity of nanoparticles by form factor modelling. However, simple geometric models such as the ellipsoid may not be enough to describe objects with complex shapes. This work shows that the form factor of gold nanobipyramids is accurately described by a truncated bicone model, which is validated by comparison with transmission electron microscopy (TEM) data for nine different synthesis batches; the average shape parameters (width, height and truncation) and the sample polydispersity are obtained. In contrast, the ellipsoid model yields worse fits of the SAXS data and exhibits systematic discrepancies with the TEM results.

## Abstract 2

The use of the bicone model is validated for extracting the form factor of gold bipyramids in solution from small-angle X-ray scattering data.

## 1. Introduction

Recent progress in materials chemistry has allowed the synthesis of nanoparticles with very well defined shape and size (Lu *et al.*, 2009 ▸). In order to stay relevant, characterization techniques must also evolve to keep up with this progress. Form factors for a wide variety of shapes have therefore been implemented in many small-angle scattering software suites (Kline, 2006 ▸; Breßler *et al.*, 2015 ▸; Doucet *et al.*, 2016 ▸; Ginsburg *et al.*, 2019 ▸; Pospelov *et al.*, 2020 ▸), but their analytical expressions can be quite complicated and the numerical evaluation very time consuming, especially since a double integral over the orientation is usually required.

An alternative (model-free) strategy consists of describing the objects as a collection of small beads (also referred to as dummy atom models, or DAMs) (Svergun, 1999 ▸). The number and positions of these ‘atoms’ are then adjusted until the scattering signal of the model approaches the experimental data. Initially developed for the study of biological macromolecules, this approach has recently been applied to in­organic nanocrystals (Burian *et al.*, 2015 ▸, 2018 ▸; Burian & Amenitsch, 2018 ▸). We do not consider these models here for two main reasons: the difficulty of converting between DAMs and geometric shapes (which are very good descriptions for the nanoparticles we are interested in) and the difficulty of accounting for polydispersity, although some progress has been made on this latter aspect (Konarev *et al.*, 2016 ▸).

A natural question to ask in this context is how detailed must the models be in order to extract as much information as possible about the morphology of the objects? Is it really useful to go beyond simple shapes, such as spheres or ellipsoids? The answer is a resounding ‘yes’ in the case of cubes. Previous work (Steiner *et al.*, 2019 ▸) has shown that the difference between cubes and rhombocuboctahedra in composite Au@Ag objects is both detectable by small-angle X-ray scattering (SAXS) and important in view of applications. In our group, we have followed by SAXS the morphological transition from spheres to cubes in such objects and confirmed these results by transmission electron microscopy (TEM) (Lyu *et al.*, 2020 ▸). A noteworthy conclusion is that both the asphericity and the polydispersity reduce the amplitude of the characteristic oscillations of the sphere form factor, but in slightly different ways: the former reduces the contrast of the fringes and preserves their number and overall profile, while the latter ‘smears’ them in the manner of a Debye–Waller factor. Although both are isometric, cubes and spheres are easily distinguished if monodisperse enough. Moreover, these shapes are instances of a more general family, that of the superball. The position of the nanoparticles along the continuum defined by the associated shape parameter can be estimated via SAXS (Dresen *et al.*, 2021 ▸) and modulates the particle packing in supercrystals (Meijer *et al.*, 2017 ▸).

Introducing anisometry (by elongating or flattening the object of interest) renders the problem more complicated, unless the resulting object has a constant section (as in a rod or plate). Since they are amenable to factorization, these limiting cases are easily treated analytically and have been extensively used in the literature. Note that factorization can also be used in the case of curved plates, considerably simplifying the calculations (Constantin, 2015 ▸).

Here, we are specifically interested in spindles, elongated objects whose section varies along the length (so factorization does not apply): are they adequately described by their equivalent ellipsoids (with an appropriate polydispersity), or would we benefit from using more realistic models? The experimental system we have investigated consists of gold nanobipyramids (Au NBPs). The synthesis of these objects has been refined over the past decade, and the interest in their optical properties and subsequent applications has grown steadily (Arenal *et al.*, 2014 ▸; Rao *et al.*, 2015 ▸; Mai *et al.*, 2021 ▸). Advanced modelling and simulations have shown how the optical response of the objects (*e.g.* the position of the surface plasmon resonance) depends on shape features such as the truncation (Liu *et al.*, 2007 ▸; Chateau *et al.*, 2015 ▸; Marcheselli *et al.*, 2020 ▸). The precise morphology of the NBPs influences their assembly in two (Shi *et al.*, 2016 ▸; Fu *et al.*, 2021 ▸) or three (Lyu *et al.*, 2022 ▸) dimensions, which in turn further modulates their optical properties.

Accurate characterization of Au NBPs is therefore imperative in view of any applications. The shape information can of course be obtained by TEM, but SAXS and other scattering techniques exhibit two major advantages: they are non-intrusive (and thus can investigate synthesis, reshaping or assembly processes) and they average over a large collection of objects (obviating the statistical issues that might affect imaging techniques). On the downside, they only yield indirect and orientation-averaged information; the comparison with TEM is of course needed for validating the models.

In this paper, we describe Au NBPs as truncated bicones. The model is used to extract morphological parameters (from both SAXS and TEM data) and these are compared with the simpler ellipsoid model. Extensive analysis of the results obtained from nine different synthesis batches shows that the bicone model is accurate enough to capture the width, height, opening angle (or, equivalently, truncation) and polydispersity of the particles, while the ellipsoid model exhibits systematic discrepancies or, in the case of the opening angle, simply does not account for this feature.

## 2. Methods

### 2.1. Materials

Gold chloride trihydrate (HAuCl_4_·3H_2_O, ≥99.9%), silver nitrate (AgNO_3_, >99%), hydrochloric acid (HCl, 37%), sodium borohydride (NaBH_4_, ≥ 96%), l-ascorbic acid (AA, ≥99%), trisodium citrate dihydrate (≥99%), cetyltri­methyl­am­monium bromide (CTAB, ≥99%), cetyltri­methyl­am­monium chloride (CTAC, 25 wt% in H_2_O) and benzyldimethylhexa­decylammonium chloride (BDAC, 99%) were purchased from Merck. Water purified by reverse osmosis with a resistivity above 15 MΩ cm was used in all experiments.

### 2.2. Bipyramid synthesis and purification

Au NBPs were synthesized as described previously (Chateau *et al.*, 2015 ▸; Sánchez-Iglesias *et al.*, 2017 ▸; Li *et al.*, 2019 ▸).

#### 2.2.1. Seed synthesis

CTAC (25 wt% in water, 2.65 ml) and water (33 ml) were heated at 303 K. HAuCl_4_·3H_2_O (25 m*M*, 400 µl) and trisodium citrate (50 m*M*, 4 ml) were then added and the mixture was kept at 303 K for 30 min. Under fast stirring, NaBH_4_ (25 m*M*, 1 ml) was added quickly. Stirring was continued for 1 min and the resulting solution was put in an oven for 5 d at 313 K prior to use.

#### 2.2.2. Growth of particle batches *A* to *D*

AgNO_3_ (10 m*M*, 2 ml), HAuCl_4_·3H_2_O (25 m*M*, 4 ml) and HCl (1 *M*, 4 ml) were added to CTAB (100 m*M*, 200 ml). AA (100 m*M*, 1.6 ml) was then added, followed by a varying quantity of seeds: 3.5, 3.5, 3.6 or 3.6 ml for samples A to D, respectively. After 4 h at 303 K, the bipyramids were centrifuged twice and purified by depletion for one night at 303 K in BDAC (350 m*M*, 15 ml) (Lee *et al.*, 2015 ▸). The supernatant was removed, and the precipitate was redispersed in water and washed twice with CTAC (1 m*M*). The Au NBPs were finally redispersed in CTAC (1 m*M*, 2 ml).

#### 2.2.3. Growth of particle batches *E* to *I*

The protocol was in all points similar to that used for samples A to D, except the solution volumes were halved and the final CTAC concentration was 2.5 m*M*. The seed volumes were 5, 2, 1, 1 and 0.1 ml for samples E to I, respectively.

### 2.3. TEM

The solutions were concentrated by slow centrifugation to a final Au^0^ concentration of 0.75 m*M* in 0.55 m*M* CTAC. A small quantity of this solution (10 µl) was then added dropwise onto a carbon-coated grid and dried at 343 K. TEM images were obtained with a JEOL 1400 microscope operating at an acceleration voltage of 120 kV.

**Figure 1:**
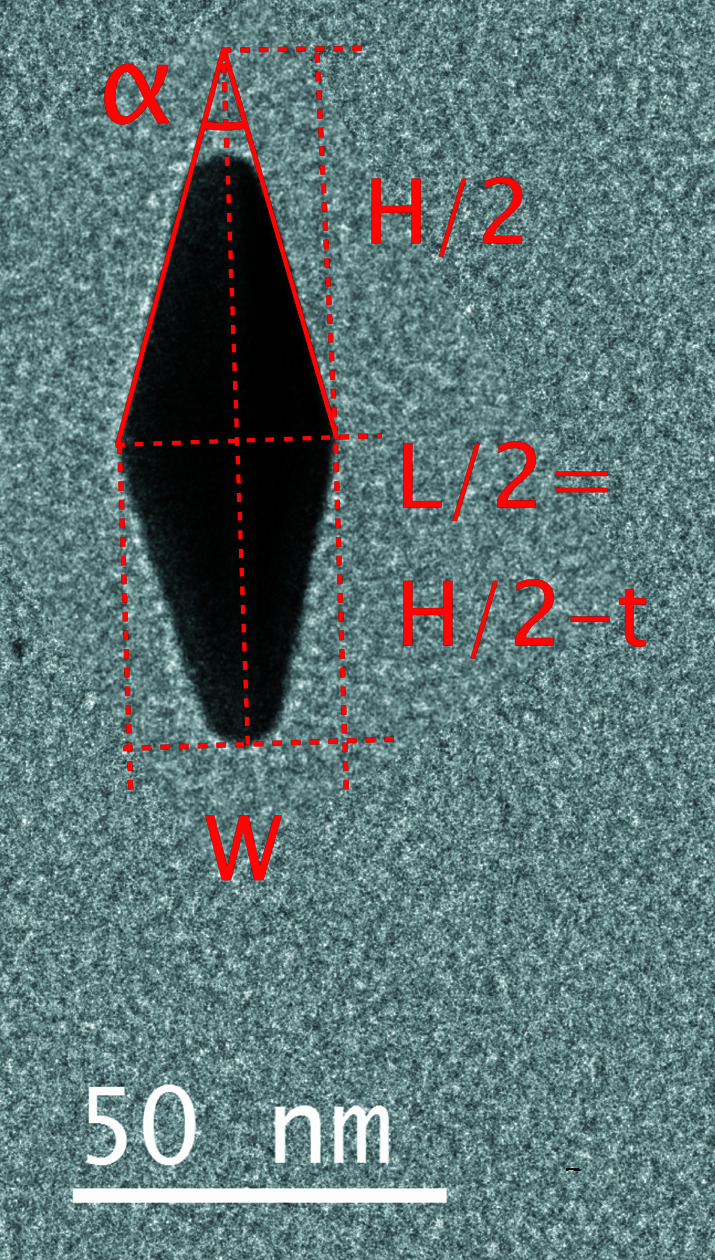
A TEM image of a particle, with the morphology parameters of the bicone model.

The bicone model is defined as in Fig. 1 ▸. The total width is denoted *W*, the total (effective) length is *L* and the total length of the bicone (without truncation) is *H*. The truncation *t* = (*H* − *L*)/2 and the full tip angle is α. These parameters are not independent, so in the following we will use the set (*W*, *L*, α) for a full description of the shape of one particle. The ellipsoid model only has two parameters, the major and minor axes *a* and *b*, respectively, which correspond to *W* and *L* of the bicone model as the length along the symmetry axis and the transverse diameter, respectively. For a complete description of the particle population in one sample we also need the polydispersity *p* (discussed below).

The TEM images are treated using *Igor Pro 7.0* (https://www.wavemetrics.com/products/igorpro). First, the particles are separated from the background using a bimodal fit: ImageThreshold operation, with the M = (2) option. The contours of each particle are then identified using the ImageAnalyzeParticles operation, with options /E/W/M = 3/FILL/EBPC. Option /E computes the equivalent ellipse for each particle, defined by the five parameters (*x*_c_, *y*_c_, *a*, *b*, θ), with *x*_c_ and *y*_c_ the coordinates of the particle centre, *a* the major semi-axis, *b* the minor semi-axis, and θ the orientation angle. They are used as a first approximation for the bicone shape (or, more precisely, for its plane projection, a truncated diamond; Fig. 1 ▸).

Both the extracted contour and the model are represented in polar coordinates [as *R*_e_(ϕ) and *R*_m_(ϕ), respectively] and the difference between them is quantified as 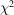 = 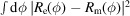 . Optimizing χ^2^ is not straightforward, but we obtained good results by a two-step approach: simulated annealing (which is more robust but does not always reach the minimum) followed by line search (to refine the parameter values further). Both steps are performed using the Optimize operation, with options M = {3, 0} and M = {0, 0} (default), respectively.

The extracted contour and the model are then presented to the user for inspection. We reject inadequately fitted contours, composite objects (where several particles are superposed and cannot be discriminated) and some round objects (possibly spheres or unreacted decahedral seeds).

### 2.4. SAXS

SAXS measurements were performed on the SWING beamline of the SOLEIL synchrotron (Saint-Aubin, France) at a beam energy of *E* = 16 keV. The sample-to-detector distance was 6.22 m, covering a scattering vector range 0.0014 < *q* < 0.24 Å^−1^. The beam size was approximately 500 × 200 µm (horizontal × vertical). All measurements were performed at room temperature (295 K). The scattered signal was recorded by an Eiger 4M detector (Dectris Ltd, Switzerland) with pixel size of 75 µm. Preliminary data treatment (angular averaging and normalization) was done using the software *Foxtrot* developed at the beamline (https://www.synchrotron-soleil.fr/fr/lignes-de-lumiere/swing), which yielded the intensity as a function of the scattering vector *I*(*q*) in absolute units. Models for the ellipsoid and the bicone were implemented in *Igor Pro 7.0*; more details are available in Appendix *A* [App appa]. Polydispersity is accounted for by a homothetic Gaussian size distribution (affecting all dimensions similarly) with relative standard deviation *p*.

## 3. Results and discussion

### 3.1. SAXS

**Figure 2:**
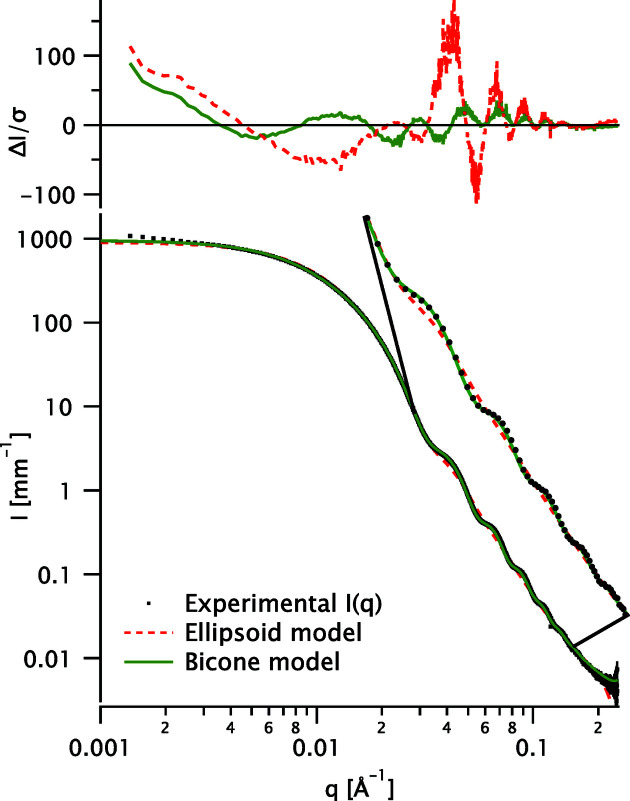
Fits to the scattering data for sample A (black dots) with the ellipsoid (red dashed line) and bicone (solid green line) models. The residuals are shown in the top panel. An enlarged view of the oscillations is shown to the right; for clarity, only one data point in ten is displayed.

**Figure 3:**
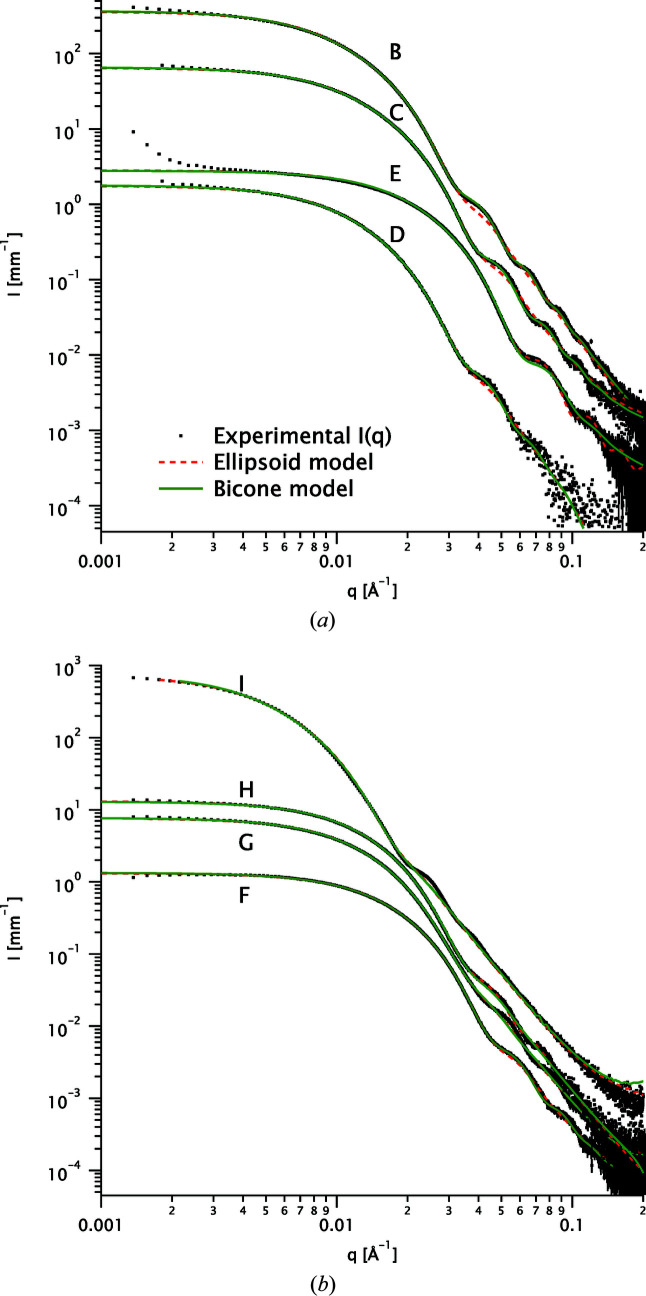
Fits to the scattering data for samples B to I (black dots) with the ellipsoid (red dashed lines) and bicone (solid green lines) models.

A detailed fit example is shown in Fig. 2 ▸ for sample A. Fits for the other samples (B–I) are shown in Fig. 3 ▸.

### 3.2. TEM

**Figure 4:**
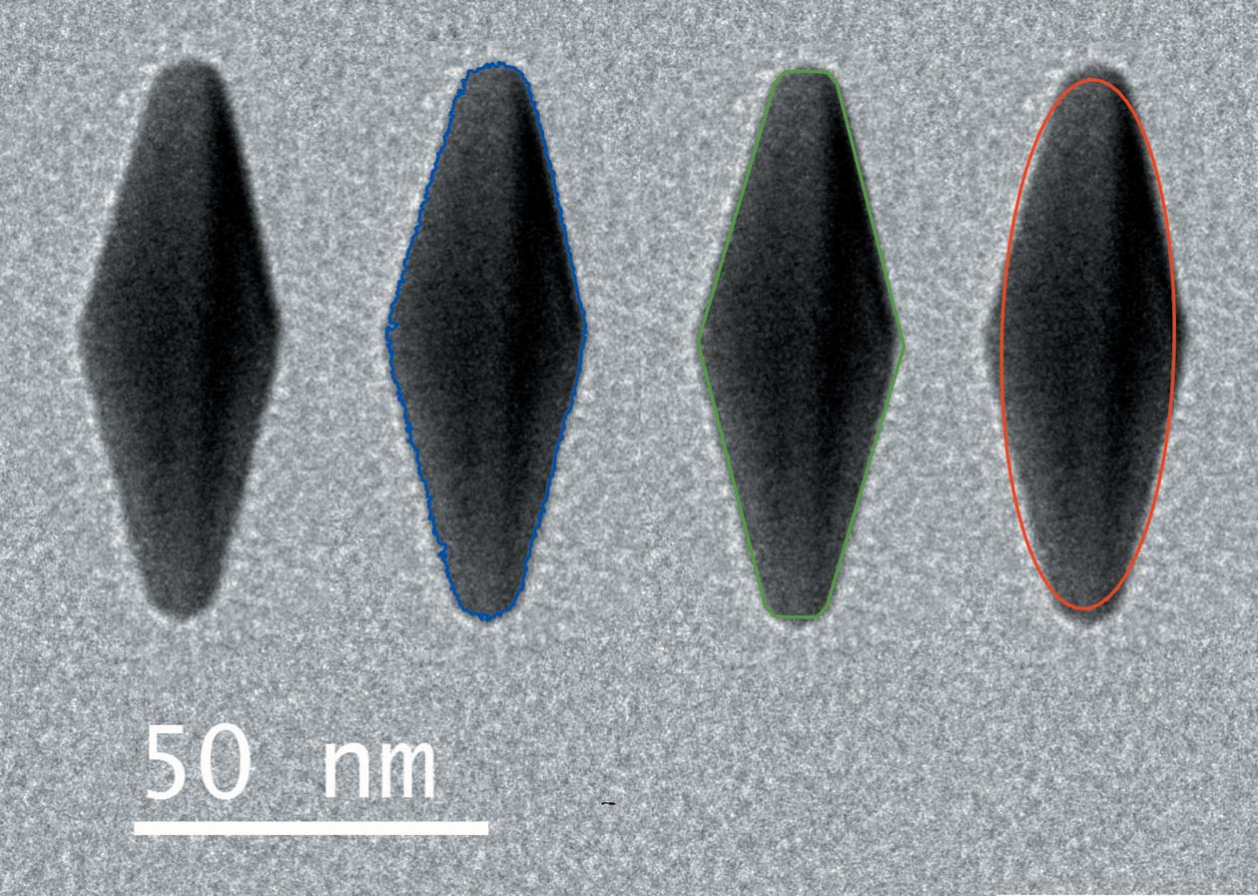
Four copies of the TEM image of one particle (from solution H). From left to right: naked image, with detected contour (blue), with bipyramid fit (green) and with ellipse fit (red).

A fit example is shown in Fig. 4 ▸ for one particle from sample H.

### 3.3. Comparison

**Figure 5:**
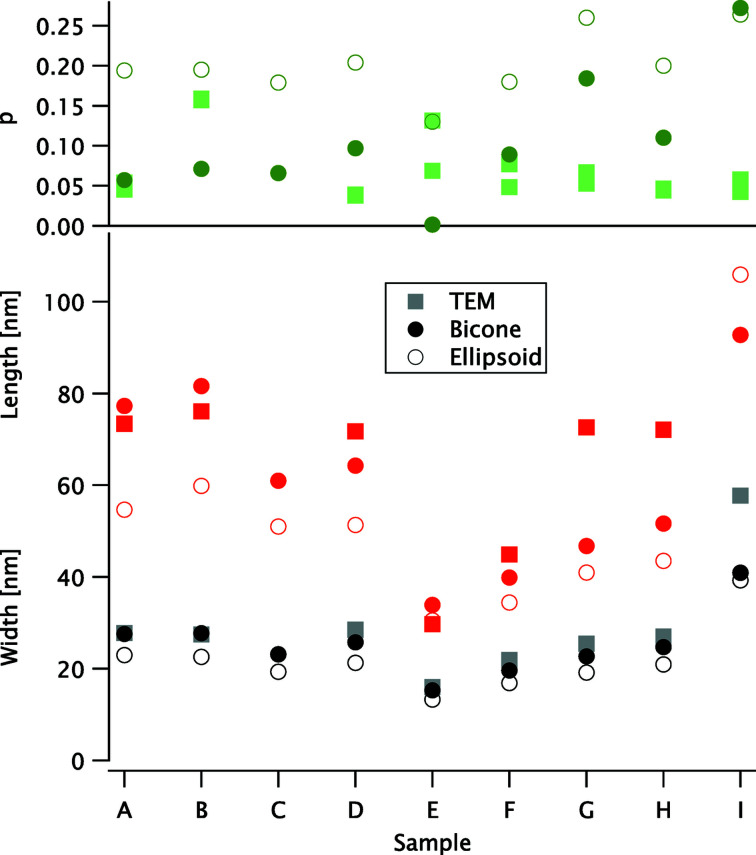
Fitting parameters for the bicone and ellipsoid models compared with the TEM values. (Top panel) Polydispersity *p* (green symbols). (Middle panel) Total length (red). (Bottom panel) Total width (black and grey). The symbols are the same for all panels: solid circles for the bicone model, open circles for the ellipsoid model and squares for the TEM data.

Three fitting parameters – the total length and width, represented by (*L*, *W*) for the bicone model and by (*a*, *b*) for the ellipsoid model, and the polydispersity *p* – can be directly compared between the two models. They are presented in Fig. 5 ▸ for all nine samples; the TEM data are also shown for comparison, except for sample C, where these data are not available. Note that the TEM analysis yields two values of *p*, as the ratio of the standard deviation to the mean value for *L* and *W*, respectively. By definition, the SAXS models only include one *p* value each.

**Figure 6:**
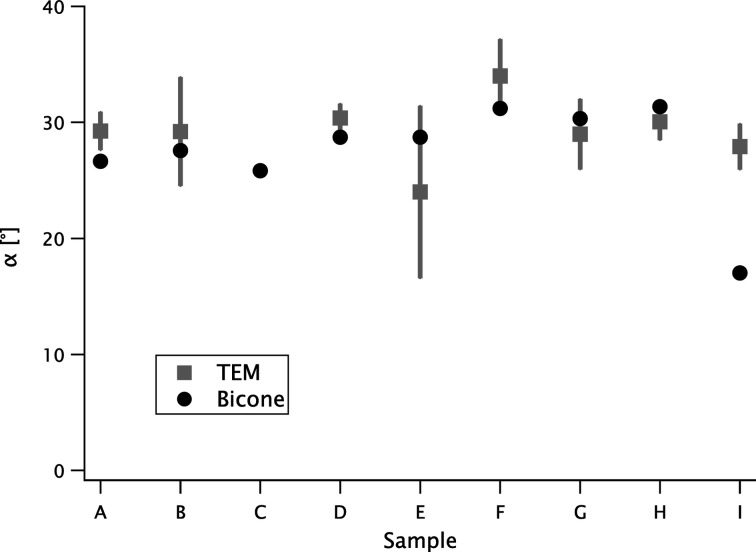
The tip angle α obtained from the bicone model (solid circles for the best fit values; error bars are smaller than the symbol size) compared with the TEM values (squares and error bars; mean ± standard deviation).

The (full) tip angle α is only accounted for by the bicone model (and, of course, by the TEM analysis). This parameter is shown in Fig. 6 ▸. Except for sample C (where the TEM data are lacking) and for sample I (where the fit quality is low), the bicone values are always within the standard deviation of the TEM distribution.

Note that the ellipsoid model cannot measure α (or, conversely, the virtual length *H*). One could of course build an angle β from the aspect ratio, *e.g.* as 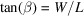 , but this is an arbitrary choice and would severely overestimate the true tip angle, because it neglects truncation: β is between 41 and 52° for all our samples.

**Figure 9:**
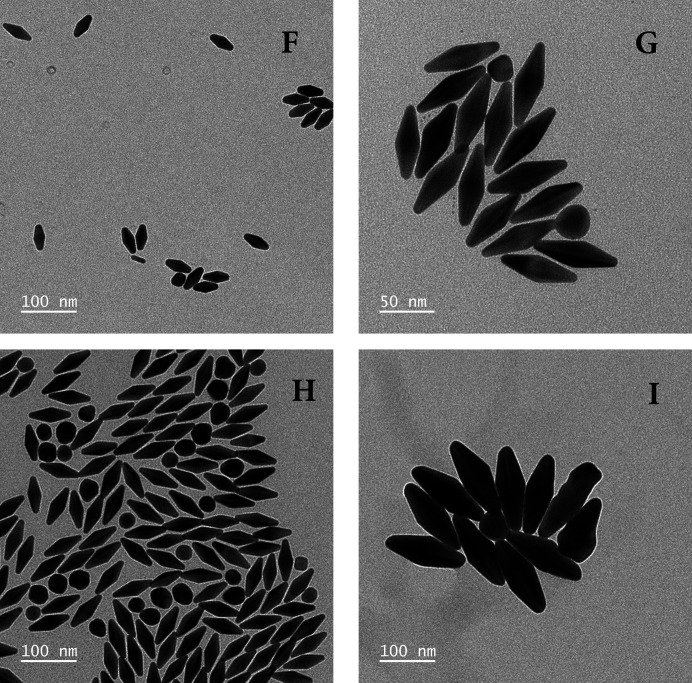
TEM images for samples F to I. The image of sample H was taken prior to purification, which explains the large number of spheres still present. Most of them were no longer present in the final sample.

All fitting parameters and some other details are given in Table 1 ▸. The absorbance spectroscopy (AS) curves are presented in Appendix *B* [App appb] and representative TEM images in Appendix *C* [App appc]. Both the bicone (BC) and ellipsoid (Ell) models yield bad fits for sample I; in particular, the polydispersity is severely overestimated (see Fig. 3 ▸ and Table 1 ▸) because the particle shape is often irregular (see Fig. 9). This is because the particle size (about 150 nm long) is at the upper limit for NBP synthesis: above it, one obtains nanojavelins (Chateau *et al.*, 2015 ▸).

## 4. Conclusions

The ellipsoid model yields reasonable values for the length and width of the objects, although they are always slightly underestimated. The polydispersity is significantly overestimated, and the tip angle cannot be inferred from this model.

On the other hand, the bicone model clearly yields much better fits to the SAXS data than the ellipsoid one, and the resulting coefficients are in very good agreement with the TEM results, in particular for the tip angle α. We conclude that this model is appropriate for describing Au NBPs. Potential applications include monitoring the growth of these objects in solution, but also the evolution of composite nanoparticles obtained by the deposition of a different metal (*e.g.* silver) onto Au NBPs (Goldmann *et al.*, 2021 ▸, 2022 ▸).

The SAXS data and the two models presented in this work, saved in the .pxp format from *Igor Pro 7.0*, are available as supporting information. The distance distribution functions for some samples are shown in Appendix *D* [App appd].

## Supplementary Material

Zip archive containing details of data and models as IgorPro output. DOI: 10.1107/S1600576722011669/jl5056sup1.zip

## Figures and Tables

**Figure 7:**
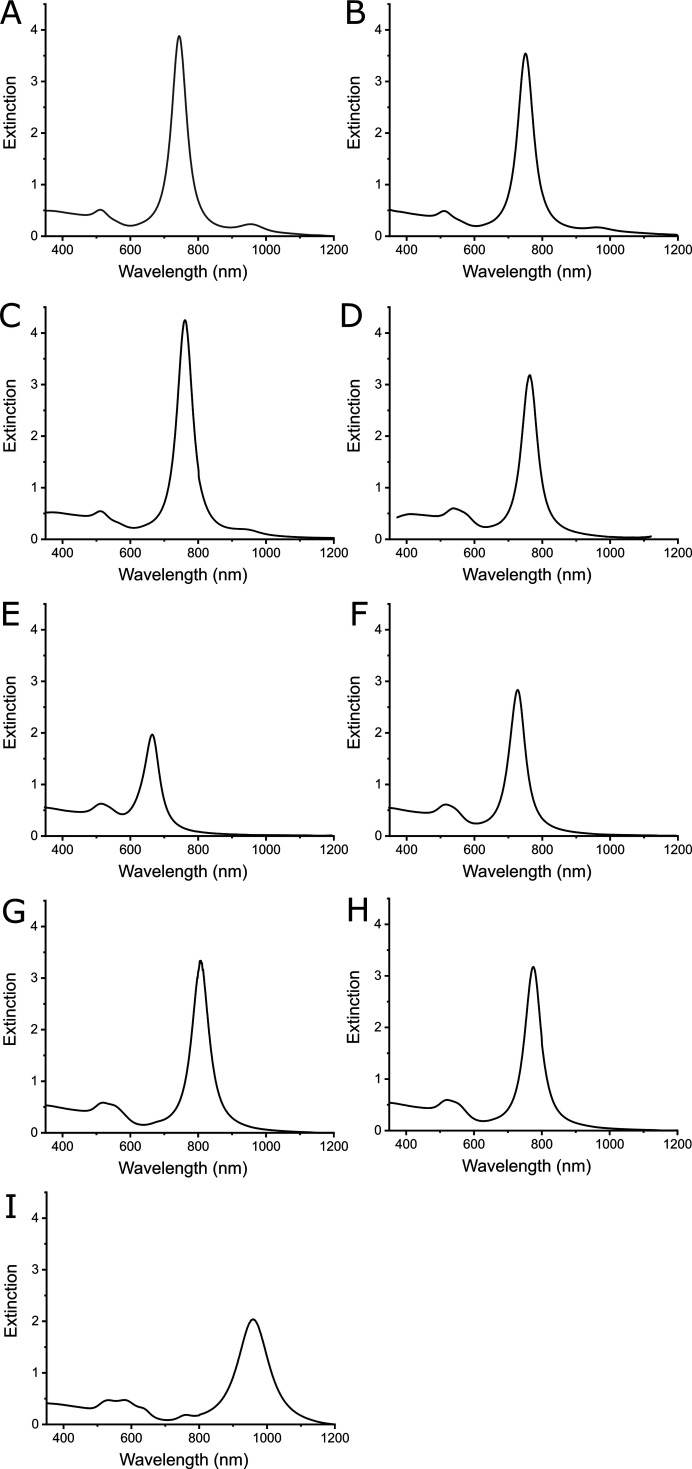
Absorbance spectra for all samples.

**Figure 8:**
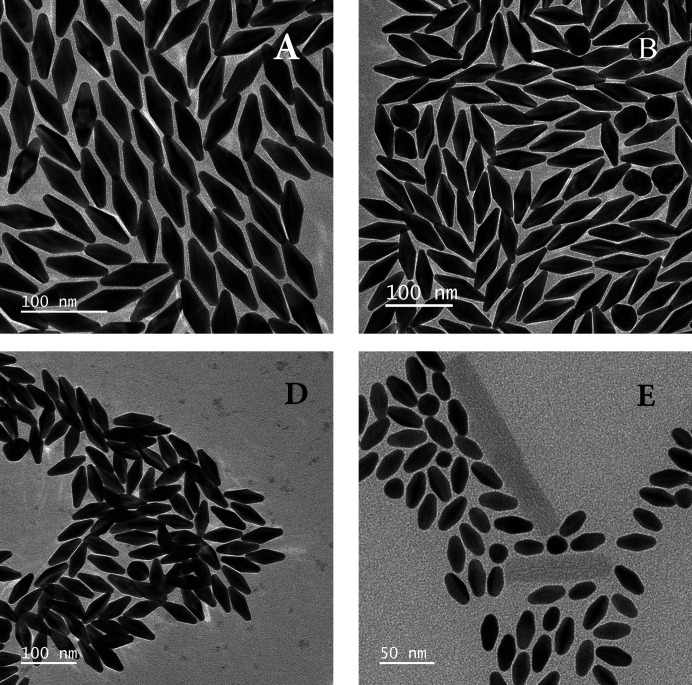
TEM images for samples A, B, D and E.

**Figure 10:**
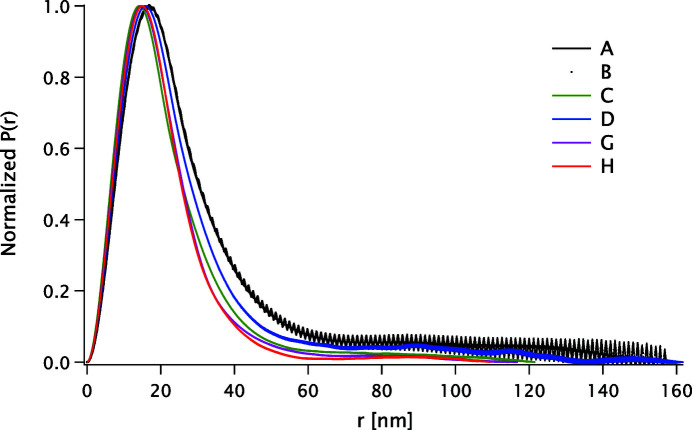
Distance distribution function *P*(*r*) for samples A, B, C, D, G and H.

**Table 1:** Parameters obtained by the three techniques (AS, SAXS and TEM) for all samples

	SAXS							TEM								AS		
Parameter	*L* (nm)	*W* (nm)	*a* (nm)	*b* (nm)	α (°)	*p*_BC_	*p*_Ell_	*L* (nm)	*W* (nm)	*a* (nm)	*b* (nm)	α (°)	*p*_*L*_	*p*_*W*_	*N*_part_ †	λ_max_ ‡ (nm)	Δλ§ (nm)	Peak ratio¶
A	77.3	27.6	54.6	22.9	26.6	0.057	0.194	73.4	27.7	72.6	23.9	29.3	0.0541	0.0454	156	743	55	7.15
B	81.6	27.7	59.8	22.6	27.6	0.071	0.195	76.1	27.5	73.5	23	29.2	0.159	0.157	47	751	57	6.75
C	61	23.1	51	19.3	25.8	0.066	0.179									759	56	7.85
D	64.2	25.8	51.3	21.3	28.7	0.097	0.204	71.7	28.5	71.2	24.3	30.4	0.0379	0.0393	31	743	56	5.23
E	33.9	15.3	30.5	13.3	28.7	0.0014	0.13	29.7	16	31.7	14.5	24	0.131	0.0685	143	668	58	3.21
F	39.9	19.6	34.4	16.9	31.2	0.089	0.18	44.9	21.9	45.7	19.1	34	0.0771	0.0482	25	731	57	4.62
G	46.7	22.7	41	19.2	30.3	0.184	0.26	72.6	25.4	70.6	21.3	29	0.0527	0.0667	39	808	60	5.55
H	51.6	24.7	43.5	20.9	31.4	0.11	0.2	72.1	27	70.5	22.9	30.1	0.0462	0.0442	67	774	58	3.6
I	92.7	40.9	106	39.3	17	0.272	0.264	153	57.7	153	49.3	27.9	0.0578	0.0424	12	957	113	5.39

† *N*_part_ is the number of particles used in the analysis.

‡ λ_max_ is the position of the longitudinal plasmon peak.

§ Δλ is the width of the longitudinal plasmon peak.

¶ The peak ratio is taken between the intensities of the longitudinal and transverse plasmon peaks.

**Table 2:** Goodness of fit χ^2^ obtained with the bicone and ellipsoid models for all samples (data and fits in Figs. 2 ▸ and 3 ▸)

Sample	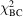	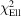
A	103.2	1112
B	14.8	51.5
C	7.4	20.5
D	2.9	4.0
E	18.0	22.7
F	3.6	8.0
G	19.8	18.6
H	4.9	8.5
I	115.8	134.2

## Appendix A Form factor models and fit quality

### A1. Ellipsoid

The ellipsoid form factor is implemented as in the NIST small-angle neutron scattering macros (Kline, 2006 ▸). Given the symmetry of the NBPs, we only consider spheroids (with major axes *a* ≠ *b* = *c*). All fits yield prolate results (with *a* > *b*).

### A2. Bicone

The form factor for a (full or truncated) cone is given in the literature (*e.g.* Renaud *et al.*, 2009 ▸) and implemented in *SASFIT* (Breßler *et al.*, 2015 ▸) and *BornAgain* (Pospelov *et al.*, 2020 ▸). For completeness, we present here its derivation for a truncated bicone, using the notation in Fig. 1 ▸. Since the body has azimuthal symmetry (around the *z* axis), we can assume without loss of generality that the scattering vector **q** is contained in the (*x*, *z*) plane, **q** = (*q*_*r*_, 0, *q*_*z*_), and makes an angle θ with the *z* axis [*q* = (4π/Λ)sinψ, where ψ is half the scattering angle and Λ is the wavelength of the incident radiation. The transverse radius of the complete cone at any height *z* between −*H* and *H* is given by

The form factor of the object is

where ρ(**r**) is the scattering length density (SLD), which depends on the position in space. Δρ is the SLD difference between the object (which is homogeneous and occupies the volume  ) and the surrounding medium. In cylindrical coordinates, the current vector is **r** = (ξ, ϕ, *z*), where the azimuthal angle ϕ is measured with respect to the *x* axis and the phase factor  =  . We can express the form factor as

For a fixed amplitude *q* = |**q**| of the scattering vector, the formula above yields the form factor for a given orientation θ. In solution, the scattering signal results from an incoherent average over all orientations, so the relevant quantity is

### A3. Polydispersity

In both the ellipsoid and bicone models we account for the polydispersity by introducing a homothetical size distribution; all dimensions are scaled by a parameter λ with respect to their reference (λ = 1) values (*W*_0_, *L*_0_, α) or (*a*_0_, *b*_0_) [in which case the scattered signal is *I*_0_(*q*)] and λ is distributed along a Gaussian,

Scaling all sizes by λ or the scattering vector *q* by the same factor preserves the signal, up to a λ^6^ prefactor (easily understood if we recall that the scattered intensity is proportional to the particle volume squared.) The polydisperse signal can then be obtained as

Relation (6) applies to any particle shape. In particular, we checked that the sphere model yields very close results to the analytical Schulz distribution as implemented in *Igor Pro* (Kline, 2006 ▸).

This algorithm has the advantage of being very fast, as it requires only one calculation of the form factor (for the reference values of the dimensions), followed by the averaging step (6). It is also very general, applying to homogeneous or composite nanoparticles of any shape. Its only disadvantage is the intrinsic limitation to homothetical polydispersity.

### A4. Fit quality

There is a significant difference in fit quality between the two models, but it is difficult to discern from the graphs in Figs. 2 ▸ and 3 ▸. We give the goodness of fit χ^2^ for both models in Table 2 ▸.

## Appendix B UV–Vis–IR absorbance spectroscopy

Absorbance spectroscopy is the most common technique for characterizing plasmonic nanoparticles. Fig. 7 ▸ presents the spectra of all nine samples, normalized to an extinction value of 0.5 at 400 nm.

## Appendix C TEM images

Figs. 8 ▸ and 9 ▸ show representative images for eight of the samples (sample C has no TEM data available).

## Appendix D Distance distribution function

In Fig. 10 ▸ are plotted the distance distribution functions *P*(*r*) for some samples, computed using the denss.f it_data routine in the *DENSS* suite (Grant, 2018 ▸). For ease of comparison, the curves are normalized to 1 at the mode. We could not obtain reliable *P*(*r*) values for curves E, F and I, presumably due to small-*q* imperfections (for the first two) and to the large particle size for the last one.
